# Pharmacological Targeting of Heme Oxygenase-1 in Osteoarthritis

**DOI:** 10.3390/antiox10030419

**Published:** 2021-03-09

**Authors:** Yohei Sanada, Sho Joseph Ozaki Tan, Nobuo Adachi, Shigeru Miyaki

**Affiliations:** 1 Medical Center for Translational and Clinical Research, Hiroshima University Hospital, Hiroshima 7348551, Japan; y.sanada3324@gmail.com; 2 Department of Orthopaedic Surgery, Graduate School of Biomedical & Health Sciences, Hiroshima University, Hiroshima 7348551, Japan; josephoz@hiroshima-u.ac.jp (S.J.O.T.); nadachi@hiroshima-u.ac.jp (N.A.)

**Keywords:** heme oxygenase-1 (HO-1), osteoarthritis, reactive oxygen species (ROS), BTB and CNC homology 1 (Bach1), nuclear factor erythroid 2 (NF-E2)-related factor 2 (Nrf2), microRNA

## Abstract

Osteoarthritis (OA) is a common aging-associated disease that clinically manifests as joint pain, mobility limitations, and compromised quality of life. Today, OA treatment is limited to pain management and joint arthroplasty at the later stages of disease progression. OA pathogenesis is predominantly mediated by oxidative damage to joint cartilage extracellular matrix and local cells such as chondrocytes, osteoclasts, osteoblasts, and synovial fibroblasts. Under normal conditions, cells prevent the accumulation of reactive oxygen species (ROS) under oxidatively stressful conditions through their adaptive cytoprotective mechanisms. Heme oxygenase-1 (HO-1) is an iron-dependent cytoprotective enzyme that functions as the inducible form of HO. HO-1 and its metabolites carbon monoxide and biliverdin contribute towards the maintenance of redox homeostasis. HO-1 expression is primarily regulated at the transcriptional level through transcriptional factor nuclear factor erythroid 2 (NF-E2)-related factor 2 (Nrf2), specificity protein 1 (Sp1), transcriptional repressor BTB-and-CNC homology 1 (Bach1), and epigenetic regulation. Several studies report that HO-1 expression can be regulated using various antioxidative factors and chemical compounds, suggesting therapeutic implications in OA pathogenesis as well as in the wider context of joint disease. Here, we review the protective role of HO-1 in OA with a focus on the regulatory mechanisms that mediate HO-1 activity.

## 1. Introduction

Osteoarthritis (OA) is the most common joint disorder that mainly affects the knee joints and is linked to an increasing socioeconomic impact owing to an growing aging population [1]. Despite its prevalence, there are currently limited treatment options available for the prevention and slowing of disease progression. OA is a complex and multifaceted whole joint disease that is characterized by articular cartilage degradation with subchondral bone sclerosis and changes in the meniscus and ligaments [2,3]. The pathological process of OA is characterized by an imbalance between receding anabolic processes and accumulating catabolic processes in the joint. Various events, such as aging and joint injury, upregulate the production of pro-inflammatory agents of oxidative stress that contribute to this imbalance.

Oxidative stress is established as a crucial factor driving age-associated diseases. The production of oxygen radicals, collectively known as reactive oxygen species (ROS), is elevated in various tissues including joint tissues with aging and diseases [4,5,6]. Oxidative stress-related imbalances between the production of ROS and the antioxidant capacity of joint cells such as chondrocytes and synovial fibroblasts has been identified as a major component of OA progression [5,7,8,9,10]. Contemporary studies, including those from our group, reported that genetically or chemically-induced antioxidant signals prevent cartilage degeneration and OA severity in aging and surgically-induced OA models [11,12,13,14,15]. Heme oxygenase-1 (HO-1), the rate-limiting enzyme in heme catabolism, is one of the most important antioxidant cytoprotective enzymes involved in the biological response to inflammation stimuli and oxidative stress. Protective functions of HO-1 were reported in numerous diseases, such as in neurodegeneration, rheumatoid arthritis (RA), and OA [10,16,17,18]. We also reported that constitutive expression of HO-1 in menisci and articular cartilage in mice reduce the severity of OA and intervertebral disc degeneration [13,19,20].

HO-1 expression and enzymatic function are mostly regulated at the transcriptional level. The principal HO-1 transcription factor nuclear factor erythroid 2 (NF-E2)-related factor 2 (Nrf2) facilitates a highly potent cellular defense response against oxidative stress by promoting the transcription of an array of genes, including HO-1, that are widely involved in redox homeostasis, xenobiotic detoxification, and metabolism [18,21]. Running counter to Nrf2, the bric-a-brac, tramtrack, and broad complex (BTB), and “cap ‘n’ collar” (CNC) homologue 1 (Bach1) are widely expressed transcriptional repressors of HO-1 belonging to the basic region leucine zipper factor family (CNC-bZIP). Under normal conditions, Bach1 and small Maf proteins form heterodimers that inhibit the transcription of the aforementioned Nrf2-regulated genes [22]. Although the main function of HO-1 is the same in both humans and rodents, previous study indicates that the mechanisms regulating their expression differs. In particular, specificity protein 1 (Sp1) and CCCTC-binding factor (CTCF) are unique to human HO-1 expression regulation [23,24]. Gene expression experiments examining Nrf2 and Bach1 implicate a clear relationship between HO-1 and OA pathogenesis in mouse models [11,13]. In addition, Nrf2/HO-1-inducible drugs have been investigated for their therapeutic potential in OA by assessing their capacity to prevent cartilage degeneration in in vitro and in vivo animal models [14,25,26,27].

In this review, we describe the most up-to-date information relating to the relationship between HO-1 activity and OA pathogenesis, and discuss the findings of drug treatment studies centered around OA prevention via HO-1 induction.

## 2. Osteoarthritis Pathogenesis and Its Relationship with Oxidative Stress

### 2.1. Osteoarthritis Development

OA is the most common form of arthritis and is known to manifest in the knee, hip, and hand joints. The condition is frequently associated with functional limitation and physical disability among the elderly [2,28], that often has severe consequences on their quality of life. Cases of OA are often classified into either primary or secondary forms (see Table 1). Primary OA is an intrinsically occurring condition caused by the aging process that universally develops in a joint gradually over a 10–15-year period. Conversely, secondary OA is characterized by traumatic and abnormal inflammatory changes localized to an area of injury in the joint caused by extrinsic risk factors, such as trauma and obesity. Age is a key risk factor for the development of OA. OA treatment is currently limited to pain management by non-steroidal anti-inflammatory drugs (NSAIDs), surgical treatment, and physical therapy, because there are no clinically approved disease-modifying osteoarthritis drugs (DMOADs) currently available [29,30]. Understanding the pathological mechanisms of OA development is needed to develop agents for OA treatment. OA pathogenesis is characterized by an imbalance between dwindling anabolic and escalating catabolic processes in the joint. Mechanistically, metabolic imbalances that manifest during OA progression are driven by numerous factors, including disintegrin-like and metallopeptidase with a thrombospondin type 1 motif 5 (ADAMTS5), matrix metalloproteinase-13 (MMP-13), various pro-oxidant factors, and hedgehog signaling [5,31,32,33,34,35]. MMP-13 and ADAMTS5 are matrix-degradation enzymes that play essential roles in OA development; they are responsible for facilitating the degradation of major extracellular matrix (ECM) components such as type 2 collagen (COL2A1) and aggrecan (ACAN) in articular cartilage [31,32,33,36].

Different mechanisms contribute to OA pathogenesis such as reduced self-renewal ability, increased production of pro-inflammatory mediators, and oxidative stress. Inflammation and oxidative stress are recognized as important risk factors in both primary and secondary OA. The inflammatory cytokines interleukin (IL)-1, IL-6, tumor necrosis factor (TNF)-α, and prostaglandin E2 (PGE2) are mediators of inflammatory states and cartilage degradation in both RA and OA [37]. In particular, IL-1β and TNF-α activate chondrocytes to produce MMP-13 and ADAMTS5 and promote catabolic conditions [38,39]. Previous studies demonstrated that IL-1β activates mitogen-activated protein kinase (MAPK) signaling pathways such as extracellular signal-regulated kinase (ERK), p38, and nuclear factor kappa-light-chain-enhancer of activated B cell (NF-κB) pathways in human articular chondrocytes [40,41,42]. These signaling pathways are well-known local and systemic activators of the inflammatory state and are therefore potent promoters of pathogenesis.

### 2.2. Oxidative Stress and Antioxidant Signaling in Joints

ROS are free radicals derived from molecular oxygen. Examples of ROS include hydroxyl radicals, hydrogen peroxide (H_2_O_2_), superoxide anions, nitric oxide (NO), and hypochlorite ions. Under normal conditions, ROS are generated by a number of typical cell functional process (such as ATP synthesis in the mitochondria) and serve as important cellular messengers and mediators of immune responses during bacterial infection [44,45]. The overaccumulation of ROS is referred to as a state of oxidative stress. In the joint, oxidative stress is induced by aging, mechanical stress, and inflammation. In OA, oxidative stress has also been associated with abnormal chondrocyte death, cellular senescence, and the expression of catabolic factors such as inflammatory cytokines and ECM-degrading proteases [46,47,48,49,50]. Accumulations of ROS activate the NOD-like receptor (NLR) family pyrin domain containing 3 (NLRP3) inflammasome in synovial membrane macrophages and increases the expression of pro-inflammatory cytokines IL-18 and IL-1β [51]. These pro-inflammatory cytokines stimulate chondrocytes and osteoclasts to activate cartilage degradation pathways, thereby driving OA pathogenesis.

Because ROS production and activity play a pivotal role in the maintenance of cellular homeostasis, mammalian cells have acquired adaptive protective mechanisms that regulate the accumulation of ROS through the production of antioxidant factors. These mechanisms have an immense influence on intracellular signaling, with studies indicating that joint cells are highly sensitive to the loss of these regulatory and control systems during OA development [12,52]. Genetic modification studies of antioxidant genes such as superoxide dismutase 2 (SOD2) and HO-1 showed that the dysregulation of antioxidative mechanisms enhances cartilage degradation, synovial inflammation, and chondrocyte senescence [6,12,53,54,55]. Moreover, Nrf2-deficiency augments cartilage injuries and oxidative damage in adjuvant-induced RA joints [56,57]. The role of Nrf2 in RA is summarized and further detailed in several other reviews [10,58]. On the other hand, Nrf2 activation and HO-1 expression using a histone deacetylase inhibitor trichostatin A (TSA) represses IL-1β-induced MMP gene expression in chondrocytes. Nrf2 acetylation is an important upstream regulatory mechanism for Nrf2 transcriptional activation [59,60]. Similarly, in vivo TSA treatment has been reported to decrease OA severity and reduce MMP expression in mice while increasing HO-1 [11]. Although HO-1 expression in knee cartilage is known to decrease with aging, our previous study using Bach1-deficient mice demonstrated that the consecutive induction of HO-1 in chondrocytes can prevent OA development in both aging and surgically-induced preclinical mouse models [13,19]. A number of reports have noted that inducers of antioxidant signals such as N-acetyl cysteine (NAC), S-allyl cysteine, and procyanidins are able to attenuate OA development induced by joint instability in mice models [61,62,63].

Collectively, these studies indicate that the imbalances in redox homeostasis caused by oxidative stress can be corrected by the appropriate application of the induction of antioxidant signals. Therefore, pharmacological modulation of antioxidant signals may represent a novel and promising strategy for the prevention of OA.

## 3. The Role of Heme Oxygenase-1 in Arthritis

### 3.1. Properties of Heme Oxygenase-1

Heme oxygenase-1 (HO-1) is a rate-limiting cytoprotective enzyme encoded by the *HMOX1* gene that functions to degrade free heme into equimolar amounts of ferrous iron (Fe^2+^), carbon monoxide (CO), and biliverdin (which is later converted into bilirubin by biliverdin reductase) [64,65,66]. It is a ubiquitously expressed [67] member of the heat shock protein family and is one of two active isozymes that make up the HO system. In contrast to its variant isozyme HO-2 (encoded by the *HMOX2* gene), HO-1 expression is inducible, whereas HO-2 is constitutively expressed and not inducible. A catalytically inactive third isoform of HO, HO-3, has also been described. HO-3 shares ~90% of its amino acid sequence identity with HO-2 [68]. Structurally, human HO-1 has a molecular weight of ~32 kDa containing 288 amino acid residues and shares a high level of sequence similarity with rodents. HO-1 is a single compact domain consisting of mostly alpha helical folds. These folds are responsible for aiding substrate orientation within the heme pocket and C-terminal, an essential domain for facilitating anchorage to the smooth endoplasmic reticulum [69].

### 3.2. Anti-Inflammatory Function of Heme Oxygenase-1

HO-1 has been shown to possess many important immunomodulatory and anti-inflammatory functions. The latest insights into the immunomodulatory functions of HO-1 have been the subject of several reviews [70,71]. A significant component of the interactions between HO-1 and the immune response is defined by its relationship with macrophages. Macrophages are an essential cellular component of the immunomodulatory system and the subsequent inflammatory response. Macrophages adopt different functional programs in response to signals from their microenvironment: they are often classified as having either a pro-inflammatory M1 (classically activated) or anti-inflammatory M2 (alternatively activated) phenotype [72]. Impairment of the balance between M1 and M2 macrophage polarization is thought to be the cause of several inflammatory-related diseases, including arthritis [72]. HO-1 expression in macrophages is dependent on stimulation by multiple transcriptional signals and cytokines. HO-1 induction was observed to direct macrophage polarization towards an M2 phenotype, thereby exerting an anti-inflammatory response [73]. Vitali et al. showed that HO-1-deficient mice-derived macrophages are more sensitive to hypoxia-induced oxidative stress, which was characterized by increased NLRP3 inflammasome signaling [70]. The elevation of HO-1 conversely attenuated complement-dependent inflammation, whereas its inhibition led to a potentiation of the inflammatory response [74]. Significantly, living and autopsy case studies have shown that HO-1-deficient mice exhibit a similar phenotype to genetically HO-1-deficient humans, such as growth retardation, anemia, iron deposition, and vulnerability to stressful injury [75,76]. In contrast, constitutively expressed HO-2-deficient mice retain an intact immune system [77]. These results indicate that inducible HO-1 carries out crucial cytoprotective functions through its immunomodulatory activities.

### 3.3. Regulation of Heme Oxygenase-1

*HMOX1* is mostly regulated at the transcriptional level, with the regulatory mechanisms underlying its expression being highly complex and often cell-specific due to the extremely diverse array of stimuli that are able to induce HO-1. For example, HO-1 induction by heme, heavy metals, growth factors, NO, oxidized lipids, and cytokines has been experimentally tested and verified [78,79]. In particular, a large number of studies have reported that the expression of HO-1 is induced by stimuli that increase intracellular levels of ROS, such as heme, heavy metals, UV light, hydrogen peroxide, and lipopolysaccharide, or by stimuli that deplete cellular glutathione stores, including buthionine sulfoximine, sodium arsenite, and iodo-acetamide [80]. The linkage of HO-1-inducible expression to oxidative stress stimuli indicates that it is a component of an adaptive cytoprotective response to environmental stressors. Furthermore, it has been shown that scavengers of ROS, such as NAC, inhibit or reduce the extent of HO-1 induction by oxidative stress [81]. These results indicate that intracellular ROS plays an important role in *HMOX1* gene expression regulation: free heme produced in response to oxidative stress is catabolized into a non-cytotoxic catabolite because oxidative stress is coupled to the induction of HO-1 [82].

#### 3.3.1. Transcriptional Regulation

*HMOX1* transcription can be induced by various signal transduction pathways that activate different transcription factors [58,83]. Given that the induction of HO-1 is tied to extracellular stimuli, its upstream mitogen-activated protein kinase (MAPK) signaling cascades such as ERK, c-Jun terminal kinase (JNK), and p38 MAPK are known to play a significant role in mediating gene expression [83,84,85,86,87]. However, the molecular regulation of HO-1 induction by stimuli is different between human and rodents [88]. Previous studies using an in vitro and humanized HO-1 transgenic mouse model revealed that several transcriptional factors such as transcription factor jun-B (JunB), Sp1, upstream stimulatory factor (USF) 1/2, and CTCF are uniquely involved in human HO-1 induction mechanisms through chromatin loop formation [23,24].

Nrf2, a 66 kDa protein and a member of the CNC-bZIP family of transcription factors, is considered to be particularly important given its crucial role for the protection of joint destruction through facilitating the induction of target genes such as NAD(P)H:quinone oxidoreductase 1 (NQO1), and, significantly, HO-1 [10,18,21,89,90]. The activity of Nrf2 is functional in a wide-ranging metabolic response to oxidative stress and constitutes a cellular sensor for oxidative stress by a nuclear shuttling mechanism with the cytosolic regulator protein Kelch-like ECH-associated protein 1 (Keap1) [91,92,93]. Kruppel-like factor 2 (KLF2), a member of the zinc finger family, has emerged as an important transcription factor in the development of OA. KLF2 expression is reduced in OA patient-derived cartilage and in IL-1β-stimulated SW1353 human chondrocytes. Genetic and pharmacological overexpression of KLF2 protects against OA progression by increasing the expression of HO-1 and NQO1 through the enhancement of Nrf2 nuclear translocation [94]. Previous studies reported that increasing KLF2 levels reduced the expression of MMP-3 and MMP-13 in monocytes, which attenuates the cartilage degradation process associated with OA [95]. Moreover, in human endothelial cells, KLF2 has been shown to play a crucial role in protecting against oxidative damage through the activation of Nrf2/HO-1 signaling [96,97].

The heme-binding protein Bach1 is a transcriptional repressor of *HMOX1* due to its competitive relationship with Nrf2 for binding to ARE [98]. During oxidative stress or increasing heme concentrations, Bach1 is displaced from ARE and exported out of the nucleus to be degraded so that Nrf2 can associate with Maf and bind to ARE sequences [98]. Thus, cellular HO-1 inducive mechanisms are tightly regulated by extracellular conditions through the described Nrf2/Keap1/Bach1 system.

#### 3.3.2. MicroRNA-Mediated Post-Transcriptional Regulation

MicroRNAs (miRNAs) are a major class of small noncoding RNAs found in animals, plants, and some viruses, which function as negative regulators of gene expression. They suppress messenger RNA (mRNA) translation by promoting their degradation via the RNA-induced silencing complex (RISC) [99,100]. miRNA expression levels are frequently altered by aging-related disorders such as OA and cancer [101,102,103,104,105]. miRNAs exist that indirectly modulate HO-1 upstream regulatory factors, such as Bach1, Nrf2, and Keap1. Eades et al. demonstrated, using miRNA-based microarray analysis, that miR-200a expression is silenced in breast cancer cells, and that ectopic re-expression increases Nrf2 nuclear translocation by directly binding to the Keap1 mRNA 3′-untranslated region (3′-UTR) and facilitating its degradation [106]. Kim et al. found that hypoxia-inducible miR-101 induces HO-1 expression in endothelial cells. Upregulated miR-101 targets the E3 ubiquitin ligase Cullin3 and stabilizes Nrf2, resulting in the enhancement of Nrf2 translocation into the nucleus [107]. On the other hand, ectopic expression of miR-28 in mammalian endothelial cells directly downregulates Nrf2 protein expression via 3′-UTR binding of Nrf2 mRNA independently from the Keap1 pathway [108]. Sangokoya et al. identified that patients of sickle cell disease highly express miR-144 in erythrocytes and abrogate antioxidant signals. miR-144 regulates Nrf2 expression through binding to the 3′-UTR of Nrf2 mRNA and modulates the oxidative stress response in K562 and primary erythroid progenitor cells [109].

miRNAs also modulate HO-1 activity by directly regulating *HMOX1* expression. Beckman et al. performed in silico analysis of the human *HMOX1*-3′ UTR and identified two candidate miRNAs, miR-377 and miR-217, as possible inhibitors of *HMOX1*. Subsequent experiments found that co-transfection of miR-377 and miR-217 downregulated the luciferase activity of *HMOX1*-3′ UTR and HO-1 protein expression levels [110]. Our previous study established that miR-140 regulates HO-1 expression by binding to the 3′-UTR of *BACH1* in human primary chondrocytes [14]. Similar investigations reported that miR-155 and miR-196a regulates the expression of HO-1 through the reduction in *BACH1* expression in endothelial cells or hepatoma cells [111,112]. miR-155 is a pro-inflammatory miRNA that is significantly upregulated in OA and RA joints [113,114,115,116]. Moreover, upregulated miR-155 inhibits the expression of a number of core proteins in the autophagy cascade and promotes the cartilage degradation pathway in chondrocytes [113]. Autophagy is a critical evolutionarily conserved eukaryotic process that functions to maintain cellular homeostasis in response to changes to the environment, including in chondrocyte protection [117,118]. While autophagic activity is decreased in aging and OA-induced mouse knee cartilage, HO-1 overexpression is able to rescue the suppression of autophagic activity [13,50]. The pro-inflammatory cytokine TNF-α induces the expression of HO-1 and miR-155 in endothelial cells [111]. In contrast, IL-1β stimuli downregulates the expression of HO-1 and miR-140 in articular chondrocytes [14]. miR-140 is highly expressed in normal cartilage but is significantly reduced in OA cartilage. miR-140 is a cartilage-specific miRNA and is the most important regulatory factor in cartilage homeostasis [102,103]. Previously, we found that carnosic acid (CA), a natural flavonoid, raises the expression of HO-1 and miR-140 in articular chondrocytes through the transcriptional downregulation of *BACH1*. In addition, we observed that CA treatment alleviates IL-1β-induced cartilage damage. These results suggest that the regulation of Bach1-mediated HO-1 expression by miRNA may depend on cell type and context.

### 3.4. Heme Oxygenase-1 in Osteoarthritis

Elevation of oxidative stress in joint tissue has long been established as a crucial factor mediating the articular cartilage degradation process during OA [5,119]. Given its antioxidant properties, several studies have suggested a protective role of HO-1 in OA pathogenesis. It has been reported that HO-1 is significantly upregulated in human and mouse models of OA, with higher levels of expression being observed in areas of cartilage damage [120,121]. However, discrepancies exist regarding Nrf2/HO-1 expression between OA cartilage and IL-1β-induced OA-like chondrocytes [122,123]. Our previous study showed that HO-1 protein expression levels in IL-1β-primed normal human primary chondrocytes are significantly decreased compared with a control [14]. Further investigation is required to determine if there is a relationship between Nrf2/HO-1 signaling and OA.

HO-1 is able to confer a protective effect in OA chondrocytes by inhibiting the pro-catabolic effects of IL-1β on the ECM components MMP-1 and MMP-13 [124,125] while reducing the production of pro-inflammatory cytokines such as TNF-α, IL-1β, IL-6, and IL-18 [126]. Conversely, HO-1 simultaneously enhances the synthesis of anabolic factors such as IGF-1, proteoglycan, and COL2A1 [127], and increases anti-inflammatory IL-10 levels [124]. A wide array of studies has investigated the protective effects of HO-1 in OA chondrocytes through its regulatory relationship with the Nrf2/Bach1 system. Nrf2 activation in OA chondrocytes has been reported to inhibit mitochondrial dysfunction, ROS production, and apoptosis induced by IL-1β [120]. Nrf2 activity also inhibits inflammation and ECM degradation in OA chondrocytes [51,94,128] and regulates cellular differentiation in chondrocytes [129,130]. Bach1 deficiency significantly elevates HO-1 expression in healthy and aged articular cartilage and reduces the severity of age-related OA-like changes [13]. Moreover, Bach1 deficiency significantly inhibits the severity of OA-like changes, such as meniscus degradation, inflammatory changes in synovium, osteophyte formation, and subchondral bone thickening [13,19]. All in all, these results suggest that HO-1 expressed in whole joint cells plays a critical role in the maintenance of joint homeostasis.

### 3.5. Heme Oxygenase-1 and Osteoarthritis-Associated Cellular Senescence

Cellular senescence is a major instigator of both aging-associated and post-traumatic OA pathogenesis [131,132]. In particular, aging-induced cellular senescence is known to be the most important risk factor for primary OA progression. As such, senolytic drugs have rapidly captured the interest of academic research and business venture as a potential avenue for attenuating OA pathogenesis and pathology [133]. Senescent cells secrete a robust pro-inflammatory secretome, known as a senescence-associated secretory phenotype (SASP). A SASP consists of a variety of cytokines, growth factors, and other soluble and insoluble material that continuously alters the structure and function of surrounding cells and tissues in a paracrine manner [134]. Joint tissues (comprising articular cartilage, subchondral bone, synovium, and infrapatellar fat pad) suffering from OA induced by aging or trauma are likely to harbor senescent cells that secrete an OA-propagating SASP [134]. In addition, although the mechanism of action is not fully understood, it is known that oxidative stress induces senescence in chondrocytes through multiple complex signaling pathways [135]. ROS can further propagate this effect by eroding protective telomeres, resulting in accelerated senescence and chondrocyte apoptosis. Several studies have shown that high shear stress alone can induce chondrocyte senescence [136,137,138].

Although still a largely unexplored avenue, a few studies have investigated the interplay between HO-1 induction and chondrocyte senescence. Principally, HO-1 has been documented to protect articular cartilage against cellular senescence. One study reported that cilostazol-induced senescence significantly attenuated HO-1 expression in human chondrocytes and, conversely, that HO-1 overexpression exerts a protective effect against cilostazol-induced senescence [53]. A follow-up study found that a regulator of HO-1 in stress-induced chondrocytes known as protein kinase casein kinase 2 (CK2) is associated with senescence in primary articular chondrocytes, and that the downregulation of CK2 induces cellular senescence by inhibiting the expression of HO-1 [139]. A separate investigation demonstrated that the expression of senescence markers such as senescence-associated β-galactosidase, p21, and caveolin1 were significantly decreased after HO-1 induction [53,140]. Taken together, while the mechanism by which HO-1 can suppress cellular senescence progression remains to be fully understood, these studies indicate that HO-1 may be a potential therapeutic target for aging-related OA development.

## 4. Pharmacological Treatment for OA Protection

### 4.1. OA Animal Models for Drug Development

Although aging has become the most common cause of OA, the mechanisms that mediate the effect of age on OA have not yet been completely elucidated. Therefore, pharmacological approaches for neither OA prevention nor disease-modification are presently available. Efficient pharmacological testing is needed to develop novel OA-modifying drugs. Many OA animal models such as those using mice, rats, rabbits, guinea pigs, and large animals have been developed to simulate human OA in pharmacological trials. These models apply various methods for inducing OA-like conditions, including aging, surgical, chemical, and genetic modifications [141,142]. Although aging-derived oxidative stress is the most important OA risk factor, most in vivo studies use either surgically-induced (such as the destabilization of the medial meniscus (DMM) method with transection of ligaments) or chemically-induced OA models [141,142,143]. Antioxidants such as NAC and procyanidins attenuate the development of OA induced by DMM surgery [61,62]. These models are useful for studying post-traumatic secondary OA, but may not be a valid method for studying the mechanisms and treatment of spontaneous aging-associated primary OA.

Spontaneous OA in mice without intervention develops much more slowly than the aforementioned induced OA models. In C57BL/6J mice, OA-like changes become detectable only by 12–18 months. Dunkin-Hartley guinea pigs are a widely used model of spontaneous aging-associated OA in the knee and other joints. Histological changes in the joints can be observed by three months of age, with disease severity increasing as time passes. Eventually, moderate to severe OA is observed around 18 months of age [144,145,146]. Although these models are advantageous, in that they are founded on pathological changes rather than post-traumatic alterations, their application is limited by the significant amount of time it takes for them to develop desirable characteristics. STR/ort mice have been established as a practically convenient mouse model of naturally occurring OA due to their relatively early onset of proteoglycan loss, articular cartilage fibrillation, ECM degradation, osteophyte formation, and subchondral sclerosis in the medial tibial condyle [147,148]. In STR/ort mice, serum levels of malondialdehyde (MDA; an oxidative stress marker) and C-terminal telopeptide of collagen type II (CTX-II; a COL2A1 degradation marker) are both higher than in control CBA mice prior to OA onset, suggesting that oxidative stress is linked to cartilage degradation [119]. Moreover, STR/ort mice tend to exhibit a higher incidence and severity of OA from 18 weeks of age, with severe lesions affecting the majority of animals by 15 months [148,149]. However, there are variations in the incidence and severity of OA within and among mouse models. The development of animal models is therefore required to further our understanding of the therapeutic efficacy of novel treatment modalities for primary OA.

In Table 2, we list OA animal models that have been used for the pharmacological testing of various antioxidant inducers. Most studies utilize methods for inducing secondary OA in animal models, such as through surgical-based DMM or anterior cruciate ligament transection (ACLT), or through chemical-based mono-iodoacetate (MIA) or papain. These animal models establish the OA phenotype in a simple, rapid, and reproducible manner. On the other hand, STR/ort mice, which are characterized by their spontaneous OA phenotype, have only been used in a single evaluation of HO-1 function in joint homeostasis via an HO-1-inducing adenovirus expression model. Furthermore, although several genetically modified mice have been developed to understand the correlation between redox signals such as Nrf2/HO-1 and OA development (see Table 3), such animal models have not yet been applied in the pharmacological study of antioxidants.

### 4.2. Identifying a Role for HO-1 from a Genetic Modification Mouse Model

A variety of genetically modified animal models that mimic the pathophysiology of OA have been developed to improve our understanding of the condition. In this way, numerous genetically modified animal models have been developed to examine the molecular mechanisms of Nrf2/HO-1 signaling in chondroprotective function (see Table 3). Nrf2-deficient mice that have undergone MIA or DMM exhibit severe cartilage degradation due to reductions in antioxidant signaling activity [11]. The use of histone deacetylase inhibitor TSA inhibits OA progression in DMM and MIA-induced OA via Nrf2 acetylation and activation of the downstream cascade. These effects are abolished by Nrf2 deficiencies in OA-induced mice. Moreover, Nrf2^−/−^ primary chondrocytes exhibited higher responsiveness to IL-1β stimuli in high glucose conditions due to the reduced expression of antioxidant genes [154]. In K/BxN spontaneous inflammatory arthritis and antigen-induced arthritis (AIA) mouse models, Nrf2 deficiencies exhibited more severe structural alterations in the joints such as synovitis, cartilage destruction, and bone erosion compared with wild type arthritic mice [56,57]. On the other hand, Nrf2 constitutively accumulated in the nuclei of Keap1-null mice, leading to the overproduction of cytoprotective target factors. Although Keap1-null mice generate high levels of cytoprotective factors, these mice exhibit severe abnormal keratinization and cornification in the esophagus and forestomach. Keap1 and Nrf2 double knock out (KO) mice reversed the aberrant phenotypic Keap1 deficiencies [156]. These results suggest that adequate Keap1-mediated Nrf2 activation and the regulation of downstream target genes exert a major protective function in OA development and joint arthritis pathogenesis.

The effects of HO-1 deficiency on pathology and pathogenesis have been evaluated in K/BxN mouse models. HO-1-deficient mice exhibit growth retardation, hepatic and renal iron accumulation, and chronic inflammation, thereby sharing a similar phenotype to genetically HO-1-deficient humans [75,76,157]. The incidence and severity of arthritis in arthritic mouse models was higher in HO-1-deficient groups compared with wild type groups [158]. The effects of the pro-inflammatory glucan zymosan on the acute inflammatory response of myeloid-specific HO-1 deficient mouse (HO-1^M-KO^ mouse) was investigated. It was found that zymosan stimulus induced higher serum inflammatory cytokine levels in HO-1^M-KO^ mice compared with wild type mice [159]. Conversely, the consecutive induction of HO-1 via Bach-1 KO protects against the development of experimental- and aging-induced OA [13]. Mild induction of HO-1 expression was ubiquitously observed in Bach-1 KO mice, including in chondrocytes, menisci, and bone marrow macrophages. Moreover, the consecutive induction of HO-1 in Bach-1 KO mice inhibited degeneration in the intervertebral disc after puncture [20]. Oxidative stress accelerates osteoclastogenesis and bone resorption. Abnormal bone resorption in the subchondral bone promotes bone deposition and leads to subchondral sclerosis. Bach-1 KO exhibited resistance to TNF-α-induced inflammatory bone loss in calvarial tissue [160]. In vitro Bach-1 KO bone marrow-derived macrophages exhibited suppressed mature osteoclast differentiation capacity, with knockdown of HO-1 partially reversing the suppressive effects of osteoclastogenesis in the presence of Bach1 deficiency. These results indicate that HO-1 has crucial roles in homeostasis, and that the consecutive mild induction of HO-1 via natural inducers may be able to prevent spontaneous OA development.

Findings from these animal studies are useful information for identifying the functions of Nrf2/HO-1 signaling in OA. However, further application of tissue-specific genetically modified mice using the Cre recombinase system is needed to better our understanding of the detailed molecular mechanisms of the Nrf2/HO-1 axis in joint homeostasis.

### 4.3. Pharmacological Treatment with HO-1

*HMOX1* expression is highly transcriptionally regulated by several injurious stress conditions such as ischemia, atherosclerosis, and inflammation [74,161]. Takeda et al. showed that the pharmacological induction of HO-1 by chemical compounds ameliorates lupus nephritis (an autoimmune disease of the kidney) by suppressing NO-dependent inflammatory responses [162]. Moreover, overexpression of HO-1 by adenovirus vector-mediated gene transfer has protective functions on lipopolysaccharide-induced lung injury [163]. These effects were produced by a systemic or local induction of HO-1. Induction of HO-1 reduces the activity of inflammatory pathways and the production of matrix degradation enzymes in in vitro OA synoviocytes and chondrocytes [54,124]. Antioxidant signal inducers such as resveratrol [126], Sauchinone [164], Licochalcone A [165,166], sinapic acid [167], Monascin [168], wogonin [169], Protandim and 6-Gingerol [122] and Nomilin [170] have all been shown to exert anti-inflammatory and chondroprotective properties in joint tissues through activation of Nrf2/HO-1 pathways both in vivo and in vitro.

Searching the following keywords (Nrf2 or HO-1) and (Osteoarthritis) in the PubMed database produced 88 articles and reviews. In this review, we selected pharmacological animal studies and categorized them based on drug name, administered method, animal model (primary or secondary), and whether Nrf2/HO-1 was expressed in the joint (see Table 4).

#### 4.3.1. Intra-Articular Injection-Based Delivery

The chosen route of administration is one of the most important factors affecting the results of in vivo animal studies. Therapeutics that can be delivered directly into the joint space offer the advantage of providing a high local concentration at the disease site while reducing the potential for adverse effects associated with systemic delivery. Corticosteroids and hyaluronate are potent anti-inflammatory agents with long-standing use in OA treatment. Intra-articular (IA) corticosteroid or hyaluronate injections are usually applied to patients who exhibit an inadequate response to initial therapy [183]. Thus, IA space injections are a safe and useful method for applying a local OA treatment.

IA injection is also able to deliver genetic modifications in specific nonvascular local areas such as cartilage. Previous studies have demonstrated that the adenovirus system effectively delivers genes to knee joint cartilage [94,184,185]. Gao et al. showed that KLF2 overexpression in SW1353 human chondrocytes protects against IL-1β-induced ROS accumulation, catabolic gene expression, and apoptosis, via activation of the Nrf2/ARE/HO-1 signaling cascade [94]. OA rat models have been established through IA injections of MIA (1 mg per cavity in 50 μL of sterile saline), an inhibitor of glycolysis. After injection of MIA on day three, either adenovirus-Klf2 (Ad-Klf2) or adenovirus-control was injected into the knee joints for three consecutive weeks (10^9^ plaque-forming units in a total volume of 10 μL). Immunohistochemical analysis revealed that MIA-injected OA rat knees featured increased quantities of TUNEL and MMP-13-positive cells in cartilage along with a dramatic reduction in KLF2 expression. Ad-Klf2 IA injection increased KLF2 expression in MIA-induced OA cartilage, which resulted in elevated Nrf2 activation in cartilage tissue and protected against proteoglycan loss. Conversely, IA injection of ML385, an Nrf2 inhibitor, significantly abrogated the KLF2-mediated suppression of apoptosis and MMP-13 elevation induced by MIA in vivo [94]. These results indicate that the KLF2/Nrf2/HO-1 axis forms a chondroprotective mechanism that induces anti-inflammation and anti-apoptosis and decreases catabolic factors in joint cartilage tissues. IA therapy applying gene modification methods might be a promising strategy for OA treatment.

Adeno-associated virus (AAV)-based gene delivery is an effective method for transducing genetic material into both systemic and local tissue, including into MIA-induced OA rat cartilage [117,186,187]. While gene therapy has been tested in human joints in clinical trials for RA and OA patients [188], the potential application of AAV-based gene transduction in spontaneous OA knee cartilage remains unclear. Kyostio-Moore et al. performed AAV-based HO-1 injections into spontaneous OA STR/ort mouse knee joints and observed robust transduction in synovial tissues and skeletal muscle, but not in cartilage. Similarly, they also found that AAV1/LacZ-treated joints exhibited β-gal activity in synovial tissue but not in cartilage [182]. Importantly, these AAV/HO-1 injections did not show an effective role for cartilage degradation in the STR/ort mouse. AAV-based gene therapy is therefore a useful tool for the delivery of treatments for arthritis and cartilage degradation. However, the efficiency of the transfer of genetic material into cartilage in spontaneous OA needs to be studied further in the context of a clinical trial.

Yang et al. have developed a new drug delivery system for targeting synovium-activated macrophages that is based on CO gas therapy [181]. CO has multifunctional anti-inflammatory functions through the regulation of numerous cellular signaling pathways including the NF-κB, toll-like receptor (TLR), and MAPK pathways [189,190,191]. CORM-401, a soluble oxidant-sensitive CO-releasing molecule, can be used to deplete H_2_O_2_ secreted by activated macrophages and elicit the responsive release of CO. To efficiently deliver CORM-401 into activated macrophages, CORM-401 was encapsulated in peptide dendrimer nanogel (PDN) carriers that were modified by folic acid (FA; a ligand of folate receptor beta) and hyaluronic acid (HA; a ligand of HA receptor (CD44)). PDN-encapsuled CORM-401 (CPHs) were injected into an MIA-induced OA rat knee joint to generate a new type of anti-inflammatory drug. Injection of MIA-induced experimental OA can easily and quickly reproduce loss of articular cartilage with severe synovitis induced by activated macrophage infiltration, ROS accumulation, and functional impairment in ratssimilar to that observed in human disease [152,153,192]. CPH treatment significantly suppressed cartilage degradation through CO release and elicited CO-induced HO-1 activation in the activated macrophage present in the synovial membrane [181]. These results indicated that targeting activated macrophages in the synovial membrane can inhibit OA progression and exert an anti-inflammatory response in joints. However, most triggers of OA pathogenesis are due to non-inflammatory changes to cartilage. As such, we must consider the function of HO-1 in the context of cartilage-specific non-inflammatory cellular protection.

Antioxidant IA injections have been shown to produce beneficial effects in joints outside the knee, such as the temporomandibular (TMJ) joint [180]. Resveratrol is a one of the most studied non-flavonoid polyphenol compounds with a stilbene structure. Resveratrol has been shown to suppress the activation of several transcription factors, including NF-κB, AP-1, and Egr-1, and to downregulate inflammatory products such as COX-2, VEGF, IL-1, IL-6, and TNF-α [193,194]. Imbalances in redox homeostasis leads to the accumulation of ROS in the cartilage, which results in the elevated secretion of pro-inflammatory cytokines due to the activity of pathological factors expressed through the activation of NF-κB signaling such as TNF-α and IL-1β [195,196]. To determine the effects of resveratrol on inflammatory damage in OA cartilage, Yulong et al. performed resveratrol IA injections in MIA-induced OA rat knee joints [126]. Resveratrol was administered to the articular space at 50 mg/kg every three days for eight weeks. Resveratrol treatment ameliorated rat OA clinical scoring, with reductions in swelling and inflammatory cytokine expression. Moreover, resveratrol treatment increased the expression of antioxidant factors SOD2 and HO-1 in joints and reduced ROS accumulation, leading to the inhibition of caspase3/9 activation in cartilage [126].

Resveratrol is well-known as a potent silent information regulator 2 type 1 (SIRT1) activator, which has crucial roles during the inhibition of NF-κB signaling in OA chondrocytes [155,197,198]. SIRT1 is associated with cartilage homeostasis and numerous cellular signaling pathways that respond to cellular energy and redox status, including anti-inflammation, senescence, apoptosis, and autophagy [197]. SIRT1 expression is decreased by aging in arthritis joint cartilage [176,179]. Genetic or chemical induction of SIRT1 suppressed inflammatory signaling and cartilage degradation, but enhanced microtubule-associated proteins 1A/1B-light chain 3B (LC3) expression and ECM synthesis [13]. Importantly, consecutive HO-1 overexpression via Bach1 knockout (Bach1^−/−^) also enhanced LC3 expression and autophagy activity in the aging cartilage. On the other hand, HO-1 siRNA transfection into primary Bach1^−/−^ chondrocytes abolished LC3 activation in IL-1β-stimulated chondrocytes [13]. These observations suggest that the resveratrol-related cartilage protective function is partially due to the SIRT1/HO-1/LC3 axis. Moreover, injecting high doses of resveratrol (100 μg once a week) into DMM-induced knee IA spaces prevents cartilage degeneration through SIRT1 induction in cartilage [178]. Thus, local HO-1 induction by resveratrol IA injection represents a promising pharmacological strategy for OA with reduced side-effects.

Because IA space injections are a safe and useful method for local OA treatment, clinicians usually select HA IA injection for inducing pain relief in OA patients. However, at a clinical level, serial IA injections for an extended period present practical and inconvenient difficulties.

#### 4.3.2. Intraperitoneal Injection-Based Delivery

There are two methods that are commonly used to model intermittent pharmacological tests: intraperitoneal (IP) injection and intragastric delivery by oral gavage (PO). PO is known to be a safe option for administering drugs because they are metabolized in the liver. Conversely, IP injection directly affects compound concentrations in serum levels, which can manifest as complications later on. Furthermore, there is an added risk of causing internal organ injury or of misplaced injection into the intestinal tract. This route of administration in animal studies is rarely used in repeated dose studies.

Sinomenine (SIN), a traditional Chinese medicinal herb, and scutellarin (SCU), a flavonoid glycoside, were administered once a day to DMM surgical model mice via IP injection. SIN IP injections were started at two weeks after surgery, while SCU IP injections were carried out immediately after surgery. These models exhibited reduced levels of cartilage destruction in DMM knee joints through the suppression of pro-inflammatory gene expression in cartilage without inducing noticeable toxic side effects [199,200]. SIN and SCU treatments enhanced Nrf2 translocation into the nucleus and increased HO-1 expression in mouse primary chondrocytes. Interestingly, SCU was able to bind to the Nrf2 binding site and activate its nuclear translocation. Peiminine (Pm), the active component of the Asian herb *Fritillaria verticillata*, was also IP-injected into DMM-induced mice. Pretreatment with Pm in IL-1β-induced mouse primary chondrocytes inhibited the expression of ECM degradation enzymes and pro-inflammatory cytokines. Moreover, Pm suppressed NF-κB signaling through the inhibition of AKT phosphorylation by activating Nrf2/HO-1 signaling in chondrocytes. In in vivo experiments, Pm treatment reduced the cartilage loss and increased Nrf2-positive chondrocyte numbers in DMM-induced OA cartilage [177]. Although the application of IP treatment in animal studies seems to be a useful method for OA prevention, its application in clinical medicine is unusual.

Non-irritating saline or phosphate buffer saline (PBS) solvents may be used to reduce IP-associated complications. Sun et al. investigated the anti-arthritic effects of saline-dissolved hyperoside (Hyp), a bioactive flavonol glycoside [201]. Hyp treatment inhibited nitric oxide synthase (iNOS), cyclooxygenase-2 (COX2), and metalloproteinase protein expression in IL-1β-induced mouse chondrocytes. IL-1β-induced ROS accumulation can induce mitochondria dysfunction, eventually leading to chondrocyte apoptosis. Hyp treatment significantly reduced ROS accumulation and protein expression of downstream pro-apoptotic factors Bcl-2-associated X (BAX), cytochrome c, cleaved caspase-9, and cleaved caspase-3. Hyp increased Nrf2 and HO-1 expression, and Nrf2 siRNA transfection partially abolished the anti-apoptotic role of Hyp treatment in IL-1β-induced chondrocytes. Moreover, Hyp prevented the phosphorylation of PI3K, NF-κB, AKT, ERK, JNK, and c-Jun. These results directly or indirectly indicate that Hyp has an anti-inflammatory role via in the inhibition of PI3K/AKT/NF-κB and MAPK signaling pathways in chondrocytes. In an in vivo study, Hyp IP injection was performed every other day in DMM-induced OA mice for four or eight weeks. Knee joint histological analysis indicated that Hyp ameliorated cartilage destruction caused by surgically-induced chondrocyte abnormalities via the enhancement of Nrf2/HO-1 expression and PI3K/AKT/NF-κB and MAPK signaling inhibition [201].

#### 4.3.3. Oral Administration

OA is an aging-associated whole joint disease, which means that the whole-body regulation of the aging process by continuous consumption of antioxidant signal inducers (antioxidants) extracted from natural compounds may represent a more efficient method for delaying OA development. Antioxidants can be orally administered by ingesting supplements and natural food sources such as fruits and vegetables. Genistein, an isoflavone extracted from soybean, was administered through standard feeding (40 mg/kg) in ACLT-induced OA rat models [172]. The compound downregulated IL-1β-induced MMPs, NOS2, and COX2 expression, while stimulating HO-1 expression in human primary chondrocytes. Cartilage degeneration in ACLT-induced OA rat knees was significantly inhibited by oral administration of genistein. Sulforaphane (SFN; 1-isothiocyanato-4-methylsulphinylbutane) is a plant-derived isothiocyanate obtained in the diet through the consumption of cruciferous vegetables (particularly broccoli) [202]. SFN protects against oxidative stress through the Nrf2-mediated induction of phase II detoxification and is known to reduce the risk of contracting various cancers. SFN inhibits the cytokine-induced expression of numerous metalloproteinases, such as MMP-1, MMP-3, ADAMTS4, and ADAMTS5, in human articular chondrocytes, fibroblast-like synoviocytes, and the SW-1353 chondrosarcoma cell line. These beneficial effects are abolished by the transfection of Nrf2 siRNA into in vitro articular joint cells. SFN-mediated activation of the Nrf2 pathway and the subsequent increase in HO-1 expression inhibits cytokine-induced MAPK and NF-κB signaling in human primary chondrocytes. To confirm these results in an in vivo murine model of cartilage destruction, an SFN-rich diet (3 μmol/day) was fed to mice that had underwent DMM. The SNF-rich diet-fed mice exhibited significantly reduced cartilage destruction at 12 weeks after DMM surgery compared to those fed a control diet [175]. Genistein and SFN are potent inducers of Nrf2/HO-1 expression in several joint cell types. Nrf2 activation enhances antioxidative signaling while inhibiting MAPKs and NF-κB signaling in joint cells. However, while genistein and SFN reduced the severity of surgically-induced OA in in vivo experiments, no significant changes to Nrf2/HO-1 signaling were observed in joint tissues.

To directly confirm drug functions in vivo, several experiments were performed using intragastric gavage administration. Chemically-induced OA models, such as papain injection into the IA space, were also used to investigate pharmacological effects [150]. Papain-induced OA knee joints exhibited severe cartilage degradation and synovitis, characterized by high levels of inflammatory immune cell invasion [203,204]. Primary rat fibroblast-like synoviocytes (OA-FLS) were isolated from papain-induced OA joints and used for in vitro study. The dihydroartemisinin derivative DC32 [(9α,12α-dihydroartemisinyl) bis (2′-chlorocinnmate)] was added into cultured OA-FLS with or without the pro-inflammatory cytokine TNF-α. DC32 suppressed the mRNA expression of pro-inflammatory cytokines as well as OA-FLS migration that normally accompanies TNF-α stimulation. To investigate the pharmacological mechanisms of DC32, MAPKs and NF-κB signaling and the Nrf2/HO-1 pathway were evaluated by Western blot and qPCR analysis. DC32 significantly inhibited the phosphorylation of ERK, but not JNK. Moreover, NF-κB signaling was also suppressed by DC32 treatment. These positive effects were abolished by Nrf2 siRNA transfection, indicating that they are tied to the Nrf2/HO-1 pathway [174]. Oral administration of DC32 in papain-induced OA models showed that the treatment ameliorates pain relief, joint swelling, and cartilage destruction when compared with a control. Chemical injection into articular space was observed to cause cartilage degradation, severe synovitis, and ROS accumulation in the synovium. Chemically-induced OA models therefore not only induce extracellular loss in cartilage, but also an RA-like inflammatory phenotype. Nrf2/HO-1-inducing compounds such as NAC and resveratrol are known to have potent anti-inflammatory functions in rheumatic synovium and OA-FLS [205,206].

Surgically-induced OA models such as DMM and ACLT are the most commonly applied method of OA induction because of the ease by which the condition can be induced [143,151]. Moracin, a natural flavonoid compound extracted from Cortex Mori (the root bark of *Morus alba*), is known for its anti-inflammatory activity via the suppression of NF-κB signaling pathway activation [207,208]. Moracin inhibits IL-1β-induced catabolic gene expression in rat primary chondrocytes through the inhibition of NF-κB signaling via Nrf2/HO1 activation. ACLT-induced OA rats were given 30 mg/kg moracin by gavage once every two days for eight weeks. Moracin administration prevented cartilage degradation and increased Nrf2- and Col2a1-positive cell numbers in cartilage tissue [173]. Piceatannol, a hydroxystilbene derived from the *Euphorbia lagascae* seeds, is present in various fruits and vegetables and has been reported to possess anti-cancerous and anti-inflammatory activities by the blocking of NF-κB signaling [209,210,211]. In human primary chondrocytes, piceatannol suppresses p65 phosphorylation through the stimulation of IL-1β, inhibits iNOS and COX2 mRNA and protein levels, and reduces NO generation and PGE2 expression in a dose-dependent manner. To investigate the protective effect of piceatannol in OA development in vivo, piceatannol (10 mg/kg in 0.5% carboxymethylcellulose) was administered intragastrically to DMM-induced OA mice once daily for eight weeks. The µCT and safranin-O staining results indicated that piceatannol treatment prevents cartilage degeneration in OA mouse models. Moreover, immunohistochemical analyses using Nrf2 and COL2A1 antibodies indicated elevated levels of Nrf2 and COL2A1 and increased ECM synthesis due to piceatannol treatment in OA cartilage [171]. Altogether, the administration of antioxidants derived from natural sources by oral gavage can prevent surgically-induced OA progression through the enhancement of Nrf2 antioxidant signaling.

Although HO-1 is one of the most important target genes regulated by Nrf2, the aforementioned studies using various antioxidant compounds do not directly indicate the molecular mechanisms of Nrf2 activation in vivo. Cai et al. showed that Nrf2/HO-1 protein expression levels in both sham and OA knee cartilage were increased by the intragastric administration of sinapic acid (SA; 10 mg/kg every two days) in DMM-induced OA mice. Moreover, SA treatment significantly decreased the mRNA expression levels of cartilage degradation enzymes, *Mmp1*, *Mmp3*, *Mmp13*, and *Adamts5*, and pro-inflammatory cytokines *Il-1*, *Il-6*, and *Tnf-α* at eight weeks after DMM surgery. The OsteoArthritis Research Society International (OARSI) scoring system was used to quantify the histopathological changes. The result showed that scores of the SA treatment group were much lower than the control group. In contrast, co-IA injection with sn-protoporphyrin (SnPP), an important HO-1 inhibitor, abolished the therapeutic effects of SA treatment [121]. These results indicate that oral administration of SA was protective against the progressive cartilage damage of osteoarthritis through HO-1 activation in knee cartilage.

Myricetin, a naturally occurring flavanol, is consumed in most diets or dietary supplements [212]. Myricetin treatment of human primary chondrocytes pre-conditioned with IL-1β inflammatory stimuli suppressed the generation of inflammatory mediators, cartilage degradation enzymes, and p65 phosphorylation. On the other hand, myricetin enhanced Nrf2 nuclear translocation and subsequent HO-1 expression in IL-1β-conditioned human chondrocytes in a dose-dependent manner. The observed chondroprotective functions were partly abolished via the co-treatment of Ly294002, a PI3K/Akt inhibitor. After DMM surgery, 20 mg/kg of myricetin was administered via intragastric administration every two days for eight weeks. The cartilage safranin-O staining area was significantly decreased in DMM mice that did not receive myricetin treatment. Conversely, myricetin-treated mice exhibited significantly improved surface structure and suppressed ECM degradation. Immunohistochemical analysis was used to evaluate the PI3K/Akt mediated Nrf2/HO-1 signaling activation in cartilage cells. In the myricetin treatment group, Nrf2 translocation to the nucleus and phosphorylated Akt expression was found to be significantly higher than in the untreated DMM group [128].

**Figure 1:**
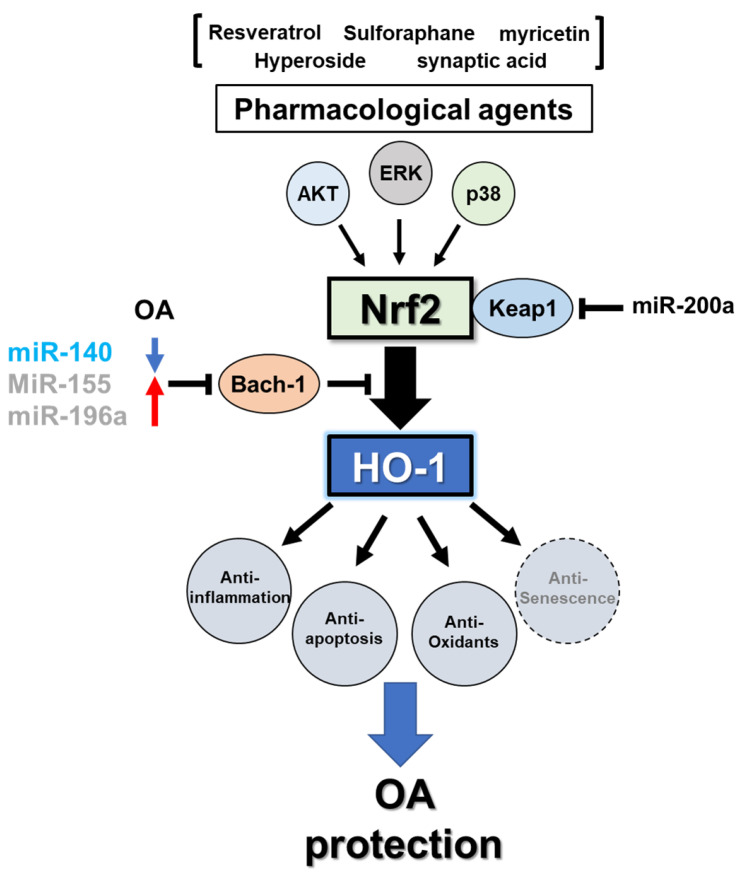
Schematic illustration of the osteoarthritis-protective mechanism of the Nrf2/HO-1 axis by pharmacological treatment of antioxidant signal inducers.

Collectively, these results suggest that the administration of antioxidants prevents OA development in OA-induced models via mechanisms that enhance Nrf2/HO-1 activation in cartilage (Figure 1). The cells involved possess similar antioxidant signaling cascades that activate Nrf2 nuclear translocation and bind to AREs upstream of *HMOX1*. Moreover, Nrf2 can directly stimulate ERK signaling to inhibit apoptosis. Therefore, to understand the critical roles of Nrf2/HO-1 signaling in spontaneous OA development, we must evaluate the precise activating location of Nrf2/HO-1 signaling in pharmacological trials using spontaneous OA animal models. Several groups, including our own, have reported that senescence accelerated mice (SAM), a rapidly aging mouse derived from the AKR/J strain, feature early spontaneous OA in the knee joints [213,214]. These animal models will be useful tools for understanding and evaluating the effective roles of antioxidants compounds. However, surgically- or chemically-induced OA pathogenesis does not sufficiently consider the characteristics of spontaneous OA development, which is uniquely caused by whole joint disabilities involving chondrocyte or bone metabolism and changes to the synovium and muscle.

## 5. Perspectives and Conclusions

OA is a global health issue marked by substantial disability and cost of medical care [1,43]. Besides contemporary surgical options (mostly through joint replacement surgery), there are no clinically available drugs that delay onset or the progression of the disease. Many age-related disabilities, including OA, are underpinned by oxidative stress and redox imbalance. Nrf2 and HO-1 are key factors that regulate redox homeostasis. The role of Nrf2/HO-1 signaling in OA has been highlighted in multiple studies, with the regulatory mechanisms of HO-1 being described as a highly promising avenue for attenuating oxidative stress at the cellular level. It is, however, unlikely that brute induction of Nrf2/HO-1 could pose as a solution; a number of reports have found evidence that permanent overactivation of Nrf2/HO-1 leads to numerous undesirable consequences, such as the dysregulation of hematopoietic regeneration [215], and neonatal jaundice [216,217]. Moreover, there are differences between HO-1 inductive mechanisms in human and rodents, making it difficult to directly apply the principles of animal studies to the clinical level. Conversely, mild HO-1 induction (3–5-fold increase in whole tissue) via Bach-1 deficiency has been documented to reduce the severity of OA-like changes in mice without debilitating effects [13]. An overall assessment of pharmacological animal studies gives the impression that a complete study of the regulatory mechanisms of HO-1 and its targets in whole joints has not yet been achieved. The link between cartilage homeostasis and Nrf2/HO-1 signaling has, to date, mostly been explored only in secondary OA animal models (see Table 1 and Table 3). A fuller picture of the detailed mechanisms explaining how HO-1 affects joint homeostasis is therefore required before potent drugs for primary OA management can be developed. In addition, understanding of the adequate level of HO-1 expression for attenuating OA pathology and pathogenesis in the whole joint including cartilage is required. While pharmacological inducers of HO-1 may be an effective therapeutic avenue for treating OA, it is more likely that clinical approaches of Nrf2/HO-1 will manifest in preventative healthcare and anti-aging therapies rather than as DMOADs. We therefore think that clinical research exploring prophylactic options (such as dietary modifications, etc.) of Nrf2/HO-1 induction is more likely to yield significant results.

## Figures and Tables

**Table 1:** Causes of osteoarthritis (OA) pathogenesis in human patients.

Risk Factor	Primary or Secondary	Mechanism
Aging	Primary	cellular senescence, mitochondrial dysfunction
Genetics	Primary	GDF5, DVWA etc, See (review [43])
Obesity	Secondary	mechanical stress, inflammatory mediators
Trauma	Secondary	joint instability, pro-inflammation
Overuse	Secondary	wear and tear, pro-inflammation
Varus and valgus alignment	Secondary	chronic overload, wear and tear

**Table 2:** List of experimental osteoarthritis animal models used in this study.

C57/B6 Mouse	Method	Time for OA Development	Mechanism	Secondary or Primary	Ref
C57/B6 mouse	DMM surgery	8 weeks	Joint instability and inflammation	Secondary OA	[143]
C57/B6 mouse	MIA injection	4~6 weeks	Inflammation and synovitis	Secondary OA	[150]
C57/B6 mouse	Papain injection	4~6 weeks	Inflammation and synovitis	Secondary OA	[150]
STR/ORT mouse	Aging	28 weeks~	Spontaneous (prone to obesity)	Primary OA	[147,148]
SD rat	ACLT surgery	8 weeks	Joint instability and inflammation	Secondary OA	[151]
SD rat	MIA injection	4~6 weeks	Inflammation and synovitis	Secondary OA	[152,153]

**Table 3:** List of antioxidants signal-related gene modification models.

Target Gene	Modification	Methods	Target Joint	OA Progression	Mechanisms	Ref
Nrf2	KO	MIA injecrtion or DMM	Knee joint	Promote	Reduction in HO-1, NQO1	[11]
Bach-1	KO	Aging or DMM	Knee joint	Prevent	mild induction of HO-1	[13]
Nrf2	KO	AIA	Knee joint	Promote	Reduction in HO-1, NQO1	[56,57]
Bach-1	KO	Aging	Meniscus	Prevent	Mild induction of HO-1	[19]
Bach-1	KO	Aging or puncture	Intervertebral disc	Prevent	Mild induction of HO-1	[20]
Nrf2	KO	IL-1β stimuli in a high glucose condition	Mouse primary chondrocyte	Higher sensitivity	Reduction in HO-1	[154]
SOD2	cKO (Col2a1 Cre)	Aging or DMM	Knee joint	Promote	Mitochondrial dysfunction	[12]
SIRT1	cKO (Col2a1 Cre)	Aging or DMM	Knee joint	Promote	Activation of NF-κB signaling	[155]

MIA (mono-iodoacetate), DMM (destabilization of the medial meniscus), AIA (antigen-induced arthritis).

**Table 4:** Pharmacological treatment for OA by Nrf2/HO-1 induction in cartilage.

Pharmacological Treatment in Secondary OA Model						
	Drug	Experimental Model	Dose and Methods	Nrf2/HO-1 Expression in Cartilage	Detection	Ref.
Oral gavage	Piceatannol	DMM mouse	10 mg/kg/day, p.o for 8weeks	Nrf2 (immunohisto) in cartilage	MMP13, Col2 (immunohisto) in cartilage	[171]
	Sauchinone	DMM mouse	10 mg/kg/day, p.o for 8weeks	-	-	[164]
	Myricetin	DMM mouse	20 mg/kg/2day, p.o for 8 weeks	Nrf2 (immunohisto) in cartilage	p-Akt (immunohisto) in cartilage	[128]
	Sinapic acid	DMM (with fat pad resection) mouse	10 mg/kg/2day, p.o for 8 weeks	HO-1 (qPCR, WB), Nrf2 (WB) in cartilage	MMP13, ADAMTS5 (qPCR) in cartilage	[121]
	Licochalcone A (Lico A)	DMM mouse	10 mg/kg/day, p.o for 8 weeks	Nrf2 (immunohisto) in cartilage	IL-1β, IL18 (ELISA)	[165,166]
	Genistein	ACLT Rat	Standard feeding with oral genistein (40 mg/kg)	-	-	[172]
	Moracin	ACLT Rat	30 mg/kg/2day, p.o for 8 weeks	Nrf2 (immunohisto) in cartilage	Col2 (immunohisto) in cartilage	[173]
	DC32 [(9α,12α-dihydroartemisinyl) bis(2-chlorocinnmate)	Papain-induced OA mouse	6.25 mg, 12.5 mg, 25 mg/kg/day, p.o for 4 weeks	-	Col2a1, MMP13 (qPCR), TNF-α (q-PCR, WB) in cartilage	[174]
	Sulforaphane	DMM mouse	feeding with AIN-93G containing 0.18 or 0.6 mg/kg	-	Col2, Col10 (immunohisto) in cartilage	[175]
	S-allyl cysteine	DMM mouse	100 mg/kg/day, p.o. for 8 weeks	Nrf2 (immunohisto) in cartilage	p16 (immunohisto) in cartilage	[63]
	hesperetin	DMM mouse	10 mg/kg/day, p.o for 8 weeks	Nrf2 (immunohisto) in cartilage	-	[27]
	Sinapic acid	DMM mouse	20 mg/kg/day, p.o for 14 days	Nrf2 (immunohisto) in cartilage	MMP13, Col2a1 (immunohisto) in cartilage	[167]
	Monascin	DMM mouse	10 mg kg/day, p.o for 8 weeks	Nrf2 (immunohisto) in cartilage	-	[168]
	Nomilin	DMM mouse	20 mg/kg/day, p.o for 8 weeks	Nrf2 (immunohisto) in cartilage	-	[170]
Intrapenetorial injection	Sinomenine	DMM mouse	10 mg/kg/day, i.p for 2 months after 1 month surgery	-	-	[176]
	Peiminine	DMM mouse	5 mg/kg/day, i.p for 8 weeks.	Nrf2 (immunohisto) in cartilage	-	[177]
	7,8-dihydroxyflavone (7,8-DHF)	DMM mouse	5 mg/kg/week, for 8 weeks	Nrf2, HO-1 (qPCR, WB) in cartilage	MMP1, 3, 13, IL-1β, TNF-α (qPCR) in cartilage	[26]
	Hyperoside	DMM mouse	20 mg/kg/2days, for 4 or 8 weeks	Nrf2 (immunohisto) in cartilage	-	[178]
	Scutellarin	DMM mouse	50 mg/kg/day, i.p for 8 weeks	-	PGE2, Cox2 (qPCR) in cartilage	[179]
Intra-articular injection	Resveratorol	MIA-induced arthritis Rat	50mg/kg/3days, for 8 weeks	Nrf2/HO-1 (WB) in joint	Cas3/9 (ELISA) in joint	[126]
	Adenovirus-KLF2	MIA-nduced OA Rat	1 × 109 PHUs/10 μL for 3 weeks	Nrf2 (immunohisto) in cartilage	Tunel staining, MMP13 (immunohisto) in cartilage	[94]
	Curcumine	Freund’s adjuvant injection TMJ OA Rat	40 μM/week, for 1 or 4 weeks	Nrf2 (immunohisto) in TMJ	MMP13, 9, IL-1β, iNOS (immunohisto) in TMJ cartilage	[180]
	FA-HA modified CORMs	MIA-nduced OA Rat	1 mg, 1.5 mg, 2.5 mg/mL/4days, for 23 days	-	TNF-α, IL-1β, IL-6 (ELISA) in joint	[181]
	Astaxanthin	DMM mouse	20 mg/kg/2 week, for 8 weeks	Nrf2 (immunohisto) in cartilage	-	[25]
Pharmacological treatment in primary OA model						
	Drug	Experimental model	Dose and Methods	Nrf2/HO-1 expression in cartilage	Detection	Ref
Intra-articular injection	rAAV/HO-1 (adenovirus)	STR/ORT OA model mouse (13–15 weeks ~25–27 week)	5 × 10^10^ drp rAAV (one shot)	HO1 in synovium (immunohisto)	β-gal staining	[182]
